# Health Disparities Scientific Research in the Division of Geriatrics and Clinical Gerontology

**DOI:** 10.1093/geroni/igab046.1403

**Published:** 2021-12-17

**Authors:** Lyndon Joseph

**Affiliations:** NIA, Bethesda, Maryland, United States

## Abstract

The Division of Geriatrics and Clinical Gerontology (DGCG) supports clinical and translational research on health and disease in the aged, and research on aging over the human lifespan, including its relationships to health outcomes. Key areas include development of new interventions for age-related conditions and pathologies, prevention and treatment of multiple chronic conditions, geriatric palliative care, factors influencing the progression of chronic diseases over the life span, and predictive markers of aging that may inform potential interventions for extension of health span. Population diversity and health disparities are critical aspects of science that cut across DGCG research areas. This presentation will highlight several examples of DGCG-supported studies related to health disparities and discuss potential future research directions. One potential upcoming research area of interest involves leveraging large data sets to examine disparities in risks and benefits of long-term osteoporosis drug therapy and drug holidays according to racial and ethnic groupings

